# Beyond mammography screening: quality assurance in breast cancer diagnosis (The QuaMaDi Project)

**DOI:** 10.1038/sj.bjc.6603506

**Published:** 2006-12-19

**Authors:** A Katalinic, C Bartel, H Raspe, I Schreer

**Affiliations:** 1 Institute for Cancer Epidemiology, University of Luebeck, Beckergrube 43-47, 23552 Lübeck, Germany; 2 Institute for Social Medicine, University Medical Centre Schleswig-Holstein, Beckergrube 43-47, 23552 Lübeck, Germany; 3 Department of Gynaecology and Obstetrics, University Medical Centre Schleswig-Holstein, Michaelisstraße 16, 24105 Kiel, Germany

**Keywords:** breast cancer, diagnosis, mammography, quality assurance, epidemiology

## Abstract

As many breast cancer cases are detected outside mammographic screening, a multidisciplinary quality management (QuaMaDi) project involving gynaecologists, double reading by radiologists. and centralised assessment, documentation, evaluation and feedback was implemented into routine breast cancer diagnosis in part of Schleswig-Holstein (Germany) with a population of 365 000 women. A cohort of 59 514 patients eligible for diagnostic mammography was examined from May 2001 to December 2005 and quality indicators, breast cancer incidence and tumour stage distribution were analysed. A total of 102 744 diagnostic processes were initiated, for 23.8% of which (24 470) a third expert reading at the reference centre was performed. Further assessment was recommended for 6.3% (6442) of all patients. In total, 1056 breast cancer cases were diagnosed (10.3 per 1000 examinations). Patients of the QuaMaDi project had a higher proportion of ‘*in situ*’ and T1 tumours (62.6% *vs* Schleswig-Holstein: 48.6%), showing that the implementation of high standards in routine diagnostic mammography can improve the quality of breast cancer diagnosis and care.

Although systematic screening programmes using mammography to detect breast cancer have been implemented and evaluated in different regions worldwide decades ago (Nystrom *et al*, 2002; Tabar *et al*, 2003), the implementation of mammography screening in Germany first began with three pilot regions in 2001 (Junkermann *et al*, 2001), and nationwide extension should be reached by 2007. But even when a systematic screening programme is established, it has to be kept in mind that it is aimed only at asymptomatic women generally aged between 50 and 69 years. Women outside this age range or with breast cancer-related symptoms are not eligible for screening programmes. These women have to undergo breast cancer diagnosis in standard care. Obviously, the diagnostic process of a screening programme according to guidelines such as the European guidelines for mammography screening (EUREF, 2006) is of an explicitly higher quality than standard breast cancer diagnosis, especially when different personnel are performing screening and diagnostic mammography (which will apply mainly for Germany). This means that patients undergoing standard breast cancer diagnosis may receive significantly lower quality diagnosis than asymptomatic women in the screening situation. The epidemiology of breast cancer shows that the problem of quality in standard breast cancer diagnosis is real and relevant. Looking at the age distribution of breast cancer, it is evident that 55% of all breast cancer cases will occur outside the screening age group (when set from 50 to 69 years) (Parkin *et al*, 2005). Taking participation rates and interval cancer rates for the screening age group into account approximately 75% of all breast cancer cases will be diagnosed in the situation of standard care.

In Schleswig-Holstein, the northernmost federal state of Germany, a process-orientated and comprehensive quality management project was implemented to improve the standard of breast cancer diagnosis (QuaMaDi=Quality assured Mammographic Diagnosis). In this article, we examine whether quality management in diagnostic breast imaging, following national and international guidelines (EUREF, 2001, 2006; Albert and Schulz, 2004) can improve the quality of breast cancer diagnosis and care.

## MATERIALS AND METHODS

### Quality-assured mammary diagnostics

The process starts with the patient's visit to the gynaecologist. This is the usual pathway for patients with breast complaints in Germany. The decision for mammography is based on a variety of factors, including clinical findings (benign or suspicious), history of breast cancer, family history of breast cancer, hormonal replacement therapy. If mammography is indicated, the patient is asked to participate in the project (with written informed consent). The resulting mammogram is categorised using the BI-RADS classification: 1=negative, 2=benign finding, 3=probably benign finding, 4=suspicious abnormality, 5=highly suspicious of malignancy (ACR, 2003). Abnormal findings are defined as BI-RADS 4 and 5. In cases of dense breast tissue (according to ACR grade III or IV), an additional ultrasound examination is performed. The mammogram and the ultrasound images are then sent to a second radiologist, who carries out an independent second reading (without knowledge of the result of the first radiologist). In cases of BI-RADS 4 or 5 or of dissent findings (BI-RADS 1/2 *vs* 3 *vs* 4/5), the documents are sent to a highly specialised breast reference centre. If further assessment is necessary, the patient is requested to visit the centre where, depending on the individual case, noninvasive or invasive examinations are performed.

### Pilot region and study population

The pilot region is located in Schleswig-Holstein (SH, 2.8 million inhabitants), the northernmost federal state of Germany. Approximately 365 000 women (293 000 older than 20 years) live in this region. Women who were members of statutory health insurance schemes (∼80% of all women) were eligible to take part in the project. Nearly all gynaecologists and radiologists in the pilot region (84 gynaecologists, 20 radiologists) and one reference centre took part in the project. Patients who were recommended a biopsy (either percutaneous or open), which was not performed in the reference centre, were followed up by the evaluation centre to assess the final diagnosis and, in case of breast cancer, the tumour stage. Cancer data (incidence and tumour stages) were compared between the pilot region and the rest of the state on a population basis using data from the regional cancer registry. For the analysis of tumour stages, cases treated with neoadjuvant therapy were excluded. Incidence rates were calculated as crude and age-standardised rates in 100 000 persons using World (WASR) standard population.

### Statistics

For descriptive statistics, we used absolute and relative frequencies for qualitative data and mean values with standard deviations (s.d.) and medians for quantitative data.

## RESULTS

From May 2001 to December 2005, the participating gynaecologists initiated 102 744 diagnostic processes among 59 514 patients (Table 1). Of all patients, 76% stated that they had undergone at least one mammography before inclusion in the project. Actual clinical findings were found at the time of the visit to the gynaecologist in 14.4% of all patients. Further demographic data are described in Table 1.

### Radiology

A total of 102 744 mammography examinations were performed. For 57.8%, an additional ultrasound was performed. The first reader interpreted 4384 (4.3%) mammography examinations as abnormal (BI-RADS 4 : 3625, 5 : 759).

For 97.6% (100 358) of all mammograms a second independent reading was carried out by another radiologist. For 811 cases expert reading was carried out directly in the reference centre, for 1516 cases second reading was not documented (for unknown reasons).

In the group of patients with BI-RADS <5 at first view (Table 2), the second reader classified 3205 additional cases (3.2% of all examinations) as abnormal. In total, 7.4% of mammograms were classified as abnormal after the second reading. Another 16 463 (16.4%) patients showed dissent findings, with BI-RADS 3 interpretation by one radiologist and BI-RADS 1 or 2 by the other (Table 2). Overall concordance of first and second reading was 77.9%. The median time lag between first and second reading was 3 days (mean 6.9±14.7). Results with BI-RADS 5 in the first reading were sent immediately to the reference centre (median time 1 day).

### Reference centre

For 23.8% (24 470) of all radiological examinations, a third reading at the reference centre was necessary because of BI-RADS 4 or 5 findings or dissent judgement between the first and second radiologist (Table 3). As a result, further assessment was recommended for 6442 cases (7.8% of these cases were judged as BI-RADS 5, 85.0% as BI-RADS 4). Referring to all examinations, the assessment rate was 6.3%.

In the reference centre, 5766 assessments were performed (89.6% of all recommended assessments). Six hundred and seventy-six assessments (10.4%) were performed elsewhere. The reference centre carried out 1540 bioptic procedures (26.7% of all assessments in the centre, 15.0 per 1000 examinations), which revealed 661 histologically verified breast cancer cases (42.9%). In the group of 121 primarily recommended surgical diagnoses, 84 (69.4%) breast cancer cases were identified; 73 cases (25.5%) were identified within the group of recommended surgical diagnosis after assessment. Within the group that had assessment elsewhere 238 breast cancer cases were identified. In total, 1056 breast cancer cases were diagnosed by the project (10.3 per 1000 examinations).

### Breast cancer incidence according to cancer registry data

In SH, 11 525 breast cancer cases (including 588 *in situ* cases) occurred between 1999 and 2003, 3201 (28%) of them in the pilot region. Age-standardised breast cancer incidence (WASR) in the pilot region increased by 7.5% after the start of the project in 2001 (1999–2000: 91.7/100 000; 2001–2003: 98.5/100 000). In the rest of the state, the incidence was stable for invasive breast cancer (1999–2000: 85.1/100 000; 2001–2003: 86.3/100 000) and for the *in situ* incidence (4.5/100 000). In the pilot region, a 100% increase of the *in situ* incidence was seen (1999–2000: 5.2/100 000; 2001–2003: 10.6/100 000).

### Tumour stage distribution

**Figure 1:**
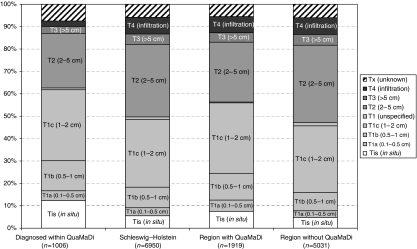
Tumour stage distribution for the QuaMaDi cohort (2001–2004), SH in total and divided into region with and without QuaMaDi (2001–2003).

Hospital discharge letters or similar information were obtained for 1006 primary breast cancer cases. Data for the T-category of the TNM stage were compared to the epidemiological population-based cancer registry data. Patients of the QuaMaDi project had a higher proportion of ‘*in situ*’ and T1 tumours than that of the whole country (62.6 *vs* 48.6%). Comparing the pilot region to SH without QuaMaDi on the basis of cancer registry data, a favourable tumour stage distribution could be found for the QuaMaDi region (pilot region: 55.0% *vs* SH without QuaMaDi: 46.0%, Figure 1).

## DISCUSSION

For a long time intensive efforts have been made to improve the quality of mammography. These efforts have dealt mainly with quality assurance in the field of mammography screening. As a result of a high compliance to technical standards, usage of diagnostic guidelines, and extensive documentation and evaluation, very high-quality mammography within the screening programmes can now be assumed (Klabunde *et al*, 2001, 2002). The positive effects of mammography screening, like reduced breast cancer mortality, have been evaluated in different programmes (Hackshaw, 2003).

In the field of diagnostic mammography quality is mainly unknown, especially if mammograms are performed in a decentralised system where a large number of radiologists are involved with different technical and educational standards. There is only a small amount of information on quality in diagnostic mammography, such as from the Breast Cancer Surveillance Consortium (BCSC) (Sickles *et al*, 2005) or from Denmark (Jensen *et al*, 2006). The requirements for diagnostic mammography are clearly lower than for screening mammography, at least in Germany. The training conditions of radiologists in the field of mammography are rather heterogeneous. There is no double reading and the standards for further diagnostics vary. A serious disadvantage is the nonexistent and/or nonstandardised documentation, which makes evaluation and audits impossible. The QuaMaDi project has taken this unsatisfactory situation into consideration and implemented multidisciplinary quality management of the existing patterns of care.

Double reading of mammograms led to a 40% increase in the most relevant group of abnormal findings (4.3% after first reading to 7.4% after second reading). This increase is higher than that reported for double reading in screening mammography. Ciatto *et al* (2005) reports a 14% increase of referral from 3.15 to 3.59% after double reading.

After the third reading, recommendation for further assessment was given for only 6.3% of the primary mammography examinations. This assessment rate is remarkably low for a diagnostic population. NHS screening reports an assessment rate of 5.8% for a cohort of women with self or general practitioner (GP) referral (which might best be comparable to our cohort) (NHS Cancer Screening Programmes, 2005). In Finland 4.6 and 2.3% of the screened women were recalled at first and subsequent screens (Sarkeala *et al*, 2004). In the Netherlands, the referral rate is only 1.38% at first screen (0.74% in subsequent screens) (Verbeek and Broeders, 2003), but here the carcinoma interval rate is very high (52%) (Fracheboud *et al*, 1999).

The biopsy rate (performed or primarily recommended) was 16.2 per 1000 examinations. It has to be emphasised that more than 90% of the recommended biopsies were percutaneous interventions. This means surgical biopsies were avoided as far as possible. As expected, the rate of biopsies in the diagnostic situation was higher than in a screening programme (Netherlands initially: 9.7/1000, subsequently: 4.7/1000; Verbeek and Broeders, 2003). Breast cancer was histologically verified in 42.9% of all biopsies performed in the reference centre. This percentage is somewhat higher than that reported for the BCSC diagnostic cohort (39.5% after biopsy) (Sickles *et al*, 2005) and only slightly lower than in the Finnish screening programme (48.7%) (Sarkeala *et al*, 2004).

In the QuaMaDi cohort, 10.3 cancer cases per 1000 examinations were found. This corresponds with the results of the self or GP referral group of the NHS screening with a breast cancer rate of 8.3/1000 women (NHS Cancer Screening Programmes, 2005). Sickles *et al* (2005) report cancer detection rates between 8 and 50 cancer cases per 1000 examinations in different diagnostic cohorts. In the screening situation, the cancer detection rates are lower, because of the lower prevalence of breast cancer in an asymptomatic population (6.5 cases/1000 in the Netherlands (Verbeek and Broeders, 2003), 4.4 and 3.6 per 1000 women (Sarkeala *et al*, 2004)).

Even though the diagnostic (process) quality in the QuaMaDi project is close to screening for many indicators, we have to question whether this also leads to a better outcome. As a surrogate parameter, tumour stage distribution could be analysed, assuming that favourable tumour stages would lead to longer survival and less morbidity. The proportion of tumours with a more favourable prognosis was 62.6% in the QuaMaDi cohort (carcinoma *in situ* was 12.3%, T1 (<=2 cm) 50.3%). These results are comparable to the diagnostic cohort described by Sickles *et al*, (2005) (20% of carcinoma *in situ*) and the self-referral/GP group of the NHS screening (17% of carcinoma *in situ*) (NHS Cancer Screening Programmes, 2005). Fracheboud *et al* (2001) report 14% carcinoma *in situ* and 64% of T1 tumours in the group of subsequently screened women.

Although QuaMaDi is not a screening programme, obvious and remarkable effects on the population-based breast cancer incidence and tumour stage distribution could be seen. After the introduction of QuaMaDi, breast cancer incidence rose by 10% in the pilot region, whereas it remained unchanged in other regions. Breast cancer incidence in the pilot region (98.5/100 000 WASR) was higher than expected for Germany (79.8), western Europe (84.6) and northern Europe (82.5) (Ferlay *et al*, 2004). A comparable increase in breast cancer incidence has also been seen in areas with screening programmes. In Norway, an increase of 13% in the female population was found comparing regions with and without screening (Zahl *et al*, 2004). The improvement of tumour stage distribution was not only found in the QuaMaDi cohort itself, but also in the whole population. This effect was seen both in the pre and post comparison of tumour stage in the pilot region (data not shown) and in the comparison within SH.

At first sight, these population-based effects of QuaMaDi are surprising, but in fact, only about 40% of all observed breast cancer cases in the QuaMaDi region (cancer registry) were recorded in the QuaMaDi project, for reasons that include the following: only 80% of the target population were members of the statutory health insurance companies, thus making them eligible for the study; informed consent was essential for participation; patients with large tumours were often sent immediately to a hospital rather than to diagnostic mammography. However, the improvement in the personal skills of the gynaecologists and radiologists and the technical improvement of the mammography equipment acquired as a result of the QuaMaDi project benefit all patients of the region regardless of their participation in the pilot project.

In conclusion, high technical and scientific standards in diagnostic mammography, including double reading of mammograms, expert reading and centralised assessment, can lead to an improved quality of structure, process and outcome in breast cancer diagnosis, even in a decentralised setting. Unnecessary biopsies, especially surgical biopsies, can be avoided and tumour stage distribution can be improved, which should lead to a better prognosis after breast cancer diagnosis. Even if QuaMaDi cannot be directly and fairly compared to mammography screening, its effects on breast cancer incidence and tumour stage distribution are similar, showing that high-quality standards in diagnostic mammography can improve breast cancer care complementary to mammography screening. As at least two-thirds of all breast cancer cases are diagnosed within standard care, detailed quality management including documentation, evaluation and feedback should be implemented for women outside the screening population. This recommendation is in accordance with the new European guidelines for quality assurance in mammography screening and diagnosis (EUREF, 2006): ‘Ethically these principles should be regarded as applying equally to symptomatic diagnostic services and screening’.

## Figures and Tables

**Table 1:** Basic demographic data

	**Diagnostic processes^a^ *N*=102 744**
*Year*	
2001	7968 7.8%
2002	20 559 20.0%
2003	23 894 23.3%
2004	24 023 23.4%
2005	26 300 25.6%
	
*Age*	
Mean and s.d.	54.9 (11.0)
Median and range	54 (18–95)
<50 years	36 282 35.3%
50–69 years	57 057 55.5%
>=70 years	9405 9.2%
	
Body mass index	25.7 (4.9)
Nulliparous	19.4%
History of breast cancer	6.3%
Family history of breast cancer	15.5%
Age at menarche (in years)	13.6 (1.6)
Menopause	50.2%
Age at menopause (in years)	48.6 (6.3)
	
*HRT*^b^ *(>1year)*	
Of all patients	38.6%
Of menopausal patients	49.9%
	
Actual clinical findings^c^	14 792 14.4%

a 59 514 patients.

b HRT=Hormonal replacement therapy.

c Any actual sign of mastodynia, induration, nipple discharge, lump, lymph node enlargement or any benign, unclear and malignant finding.

**Table 2:**
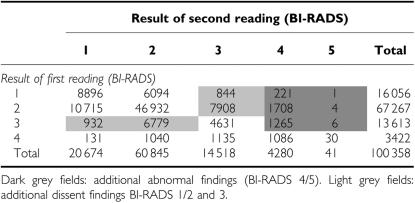
Radiological examination – results of second reading after a result of BI-RADS <5 at first reading: grey fields represent additional cases for expert reading in the reference centre

**Table 3:** Reference centre: expert reading, assessment and tumour findings

**Expert reading**	**24 470 (23.8% of all examinations)**
Time between second and expert reading (days)^a^	14.7 (10.4) 13.0
	
*Result*	
No breast cancer (radiological)	17 827 72.9%
Assessment in reference centre recommended	6442 26.3% (6.3% of all examinations)
Open surgical biopsy recommended	121 0.5%
Other^b^	80 0.3%
	

**Assessments performed in the reference centre**	**5766 (89.6% of recommended assessments)**
Time between expert reading and assessment (days)^a^	30.8 (26.3) 26.0
	
*Percutaneous interventions performed*	1540 (26.7% of performed assessments)
Histologically verified breast cancer	661 42.9%
Benign findings	879 57.1%
	
*Final assessment result*	
No breast cancer (clinical and radiological)	4714 81.8%
Histologically verified breast cancer	661 11.5%
Open surgical biopsy recommended	286 5.0%
Other^c^	105 1.8%
	
*Tumour findings*	
Total findings	1056 (10.3 per 1000 examinations)

a Mean, standard deviation and median.

b 20 missing cases, 57 cases where mammography had to be repeated, 1 case with no further diagnosis (metastases), 2 cases lost to follow up.

c 31 missing cases, 74 cases with need of further non operative diagnosis but lost to follow up.

## References

[bib1] ACR (2003) Breast Imaging Reporting and Data System, Breast Imaging Atlas. Reston, VA: American College of Radiology

[bib2] Albert US, Schulz KD (2004) Short version of the Guideline: Early Detection of Breast Cancer in Germany. An evidence-, consensus-, and outcome-based guideline according to the German Association of the Scientific Medical Societies (AWMF) and the German Agency for Quality in Medicine (AeZQ). J Cancer Res Clin Oncol 130: 527–536 15221468 10.1007/s00432-004-0558-7 PMC12161867

[bib3] Ciatto S, Ambrogetti D, Bonardi R, Catarzi S, Risso G, Rosselli DT, Mantellini P (2005) Second reading of screening mammograms increases cancer detection and recall rates. Results in the Florence screening programme. J Med Screen 12: 103–106 15949122 10.1258/0969141053908285

[bib4] EUREF (2001) European Guidelines for Quality Assurance in Mammography Screening. Luxembourg: European Commission

[bib5] EUREF (2006) European Guidelines for Quality Assurance in Mammography Screening and Diagnosis. Luxembourg: European Commission

[bib6] Ferlay J, Bray F, Pisani P, Parkin DM (2004) GLOBOCAN 2002: Cancer Incidence, Mortality and Prevalence Worldwide IARC Cancerbase no. 5. version 2.0. Lyon: IARCPress

[bib7] Fracheboud J, de Koning HJ, Beemsterboer PM, Boer R, Verbeek AL, Hendriks JH, van Ineveld BM, Broeders MJ, de Bruyn AE, van der Maas PJ (1999) Interval cancers in the Dutch breast cancer screening programme. Br J Cancer 81: 912–917 10555768 10.1038/sj.bjc.6690786 PMC2374303

[bib8] Fracheboud J, de Koning HJ, Boer R, Groenewoud JH, Verbeek AL, Broeders MJ, van Ineveld BM, Hendriks JH, de Bruyn AE, Holland R, van der Maas PJ (2001) Nationwide breast cancer screening programme fully implemented in The Netherlands. Breast 10: 6–11 14965550 10.1054/brst.2000.0212

[bib9] Hackshaw A (2003) EUSOMA review of mammography screening. Ann Oncol 14: 1193–1195 12881375 10.1093/annonc/mdg321

[bib10] Jensen A, Vejborg I, Severinsen N, Nielsen S, Rank F, Mikkelsen GJ, Hilden J, Vistisen D, Dyreborg U, Lynge E (2006) Performance of clinical mammography: a nationwide study from Denmark. Int J Cancer 119: 183–191 16450388 10.1002/ijc.21811

[bib11] Junkermann H, Becker N, Peitgen HO (2001) Concept and implementation of model projects for mammography screening in Germany. Radiologe 41: 328–336 11388053 10.1007/s001170051010

[bib12] Klabunde C, Bouchard F, Taplin S, Scharpantgen A, Ballard-Barbash R (2001) Quality assurance for screening mammography: an international comparison. J Epidemiol Community Health 55: 204–212 11160176 10.1136/jech.55.3.204 PMC1731857

[bib13] Klabunde CN, Sancho-Garnier H, Taplin S, Thoresen S, Ohuchi N, Ballard-Barbash R (2002) Quality assurance in follow-up and initial treatment for screening mammography programs in 22 countries. Int J Qual Health Care 14: 449–461 12515331 10.1093/intqhc/14.6.449

[bib14] NHS Cancer Screening Programmes (2005) Annual Review 2004. Patnick J: Sheffield

[bib15] Nystrom L, Andersson I, Bjurstam N, Frisell J, Nordenskjold B, Rutqvist LE (2002) Long-term effects of mammography screening: updated overview of the Swedish randomised trials. Lancet 359: 909–919 11918907 10.1016/S0140-6736(02)08020-0

[bib16] Parkin DM, Whelan SL, Ferlay J, Storm H (2005) Cancer Incidence in Five Continents, Volumes I to VIII. Lyon: IARC Scientific Publication

[bib17] Sarkeala T, Anttila A, Forsman H, Luostarinen T, Saarenmaa I, Hakama M (2004) Process indicators from ten centres in the Finnish breast cancer screening programme from 1991 to 2000. Eur J Cancer 40: 2116–2125 15341987 10.1016/j.ejca.2004.06.017

[bib18] Sickles EA, Miglioretti DL, Ballard-Barbash R, Geller BM, Leung JW, Rosenberg RD, Smith-Bindman R, Yankaskas BC (2005) Performance benchmarks for diagnostic mammography. Radiology 235: 775–790 15914475 10.1148/radiol.2353040738

[bib19] Tabar L, Yen MF, Vitak B, Chen HH, Smith RA, Duffy SW (2003) Mammography service screening and mortality in breast cancer patients: 20-year follow-up before and after introduction of screening. Lancet 361: 1405–1410 12727392 10.1016/S0140-6736(03)13143-1

[bib20] Verbeek AL, Broeders MJ (2003) Evaluation of The Netherlands breast cancer screening programme. Ann Oncol 14: 1203–1205 12881378 10.1093/annonc/mdg324

[bib21] Zahl PH, Strand BH, Maehlen J (2004) Incidence of breast cancer in Norway and Sweden during introduction of nationwide screening: prospective cohort study. BMJ 328: 921–924 15013948 10.1136/bmj.38044.666157.63 PMC390204

